# Letter to the editor regarding: risk factors for axial symptoms following laminoplasty for cervical spondylotic myelopathy

**DOI:** 10.1186/s13018-023-04508-8

**Published:** 2024-01-03

**Authors:** Milad Babaei Guilan, Ehsan Alimohammadi

**Affiliations:** Department of Neurosurgery, Kermanshah University of Medical Sciences, Imam Reza Hospital, Kermanshah, Iran

## Dear Editor,

We are writing to bring attention to an important study that sheds light on the risk factors associated with the development of axial symptoms (AS) after laminoplasty, a commonly used surgical procedure for treating cervical spondylotic myelopathy (CSM). The study, titled "Risk Factors for Axial Symptoms Following Laminoplasty for Cervical Spondylotic Myelopathy [1]," provides valuable insights into the factors that contribute to the occurrence of AS and highlights potential preventive measures. The authors conducted a retrospective study involving 264 patients who underwent laminoplasty for CSM between January 2018 and January 2022. The patients were evaluated based on the occurrence of postoperative axial symptoms and were divided into two groups: an AS group and a non-AS group. Various factors, including demographic information, surgical-related data, and imaging data, were analyzed to identify the risk factors and potential protective factors associated with AS. The study revealed several significant findings. High preoperative anterior spinal canal occupation rate, intraoperative facet joint destruction, C7 spinous process muscle stop point damage, larger angle of laminar opening, and greater postoperative cervical curvature loss and cervical range of motion loss were identified as independent risk factors for the development of AS. On the other hand, a larger preoperative cervical curvature and early postoperative functional exercises were shown to be protective factors against AS.

These findings have important implications for clinical practice. Surgeons performing laminoplasty can utilize this information to assess the risk of postoperative axial symptoms in patients with CSM. Preoperative evaluation of the anterior spinal canal occupation rate and cervical curvature can help identify individuals who are at higher risk of developing AS. Additionally, implementing early postoperative functional exercises may reduce the occurrence of AS.

The study's results contribute to our understanding of the complications associated with laminoplasty for CSM and provide valuable insights for optimizing patient outcomes. By identifying risk factors and protective measures, clinicians can make informed decisions regarding patient selection, surgical technique, and postoperative care.

While the study provides valuable insights, it is important to acknowledge certain limitations:

1. Retrospective Design: The study utilized a retrospective design, which inherently introduces limitations in data collection and potential biases. The reliance on existing medical records and patient recall may lead to incomplete or inaccurate information.
2. Single-Center Study: The study was conducted at a single institution, which may limit the generalizability of the findings. Different centers may have variations in surgical techniques, patient populations, and postoperative management, which could impact the results.
3. Sample Size: Although the study included 264 patients, a larger sample size could provide more robust and generalizable results. A larger sample would increase the statistical power and enhance the reliability of the findings.
4. Lack of Randomization: The study did not employ randomization, which could introduce selection bias. The assignment of patients to the AS and non-AS groups may have been influenced by various factors, potentially affecting the results.
5. Missing Data: In any retrospective study, missing data can be a limitation. The study may have encountered incomplete records or missing data points, which could impact the accuracy and comprehensiveness of the analysis.
6. Potential Confounding Factors: While the study aimed to identify risk factors for axial symptoms, there may be other confounding factors that were not considered or adequately controlled for. Factors such as comorbidities, smoking status, and occupational factors could influence the occurrence of axial symptoms but were not included in the analysis.
7. Lack of Long-term Follow-up: The study had a follow-up period of an average of 19.5 months, which may not capture the long-term outcomes and potential late-onset axial symptoms that could arise beyond this timeframe.
8. Subjective Assessment of Axial Symptoms: The assessment of axial symptoms may rely on subjective measures, such as patient-reported outcomes or clinical evaluation. This subjectivity introduces the possibility of bias and variability in the interpretation of symptoms.
9. Limited Scope of Parameters: The study focused on specific parameters such as preoperative cervical curvature, intraoperative factors, and postoperative cervical changes. Other potential factors contributing to axial symptoms, such as patient characteristics, psychological factors, and surgical complications, were not included in the analysis.
10. Lack of Control Group: The study did not include a control group of patients who did not undergo laminoplasty. A comparison with such a group would have provided a better understanding of the specific contribution of laminoplasty to the development of axial symptoms.

In another study titled "Analysis of Risk Factors of Axial Neck Pain in Posterior Cervical Single-Door Laminoplasty from the Perspective of Cervical Sagittal Plane [2]" the authors explored the potential relationship between cervical sagittal parameters in radiological images and the occurrence of axial neck pain (ANP) in patients who have undergone posterior cervical single-door laminoplasty. The study, conducted by Zuo et al., involved 141 patients who underwent posterior cervical single-door laminoplasty between January 2018 and January 2021. Among the participants, 38 were enrolled in the ANP group, while 103 were enrolled in the non-ANP group. Radiological parameters, including C2–7 Cobb angle, C2–7 sagittal vertex axis (SVA), thoracic inlet angle, neck tilt, and T1 slope, were measured using computed tomography. Statistical analyses, including Spearman correlation tests and logistic regression, were performed to explore correlations and identify potential risk factors for the occurrence of ANP.

The study's findings indicate that C2 involvement and greater T1 slope were independent risk factors for the development of axial neck pain in patients who underwent laminoplasty of the cervical spine. The results also revealed statistically significant differences in T1 slope and C2–7 SVA between the ANP and non-ANP groups. Moreover, patients with C2 spinous process involvement demonstrated more severe ANP symptoms compared to those in the non-ANP group.

The study's methodology and findings contribute valuable insights into understanding the factors associated with the occurrence of axial neck pain following posterior cervical single-door laminoplasty. By identifying C2 involvement and greater T1 slope as independent risk factors, this research provides important information for surgeons in assessing and managing patients' postoperative outcomes.

It is noteworthy that this study has some limitations. The sample size may be relatively small, potentially limiting the generalizability of the findings. Additionally, the study's retrospective design may introduce biases and limitations inherent to this type of research. Further studies with larger sample sizes and prospective designs are warranted to validate these findings and strengthen the evidence in this area.

In the recently published study conducted by Zuchang Li et.al [3], the authors aimed to explore and analyze the risk factors associated with axial symptoms after posterior cervical laminoplasty. The study conducted a retrospective follow-up of patients who underwent posterior cervical laminoplasty at Jishuitan Hospital between May 2005 and July 2011. The patients included in the study had multi-segmental cervical stenosis or cervical ossification of the posterior longitudinal ligament. Various factors, including patients' demographics, preoperative symptoms, medical complications, surgical variables, and postoperative outcomes, were collected and analyzed using statistical methods. The findings of the study are significant, as they shed light on the risk factors associated with axial symptoms following posterior cervical double door laminoplasty. The authors reported that younger age, less preoperative C2-C7 Cobb extension, operation-induced destruction of the C7 spinous process muscle stop point, higher intraoperative blood loss, and lower postoperative best JOA score were identified as risk factors for the development of axial symptoms. Additionally, the study found that the diagnosis of ossification of the posterior longitudinal ligament, older age, and greater preoperative C2-C7 Cobb extension were protective factors against the occurrence of axial symptoms. The study's methodology and comprehensive analysis of various factors contribute to our understanding of the postoperative outcomes of posterior cervical double door laminoplasty. By identifying these risk factors, the study provides valuable insights for surgeons in predicting and managing axial symptoms in patients undergoing this surgical procedure. However, it is important to acknowledge certain limitations of the study. The retrospective nature of the research design and the relatively small sample size may restrict the generalizability of the findings. Additionally, factors not accounted for in the study, such as patient lifestyle and comorbidities, may have influenced the occurrence of axial symptoms. In conclusion, the study on risk factors for axial symptoms following laminoplasty for cervical spondylotic myelopathy provides valuable insights into the factors influencing the occurrence of axial symptoms after the surgical procedure. The findings emphasize the importance of preoperative evaluation, including assessing the anterior spinal canal occupation rate and cervical curvature, in identifying patients at higher risk of developing axial symptoms. Additionally, the study highlights the significance of surgical techniques, such as minimizing facet joint destruction and C7 spinous process muscle stop point damage, to reduce the risk of axial symptoms. Furthermore, the study suggests that postoperative care, particularly early implementation of functional exercises, can play a protective role in preventing axial symptoms. These findings have important implications for clinical practice, as they enable surgeons to make informed decisions regarding patient selection, surgical approach, and postoperative management to enhance patient outcomes and minimize complications.

## Data Availability

Not applicable.

## References

[1] Ruan C, Jiang W, Lu W, Wang Y, Hu X, Ma W (2023). Analysis of risk factors for axial symptoms after posterior cervical open-door laminoplasty. J Orthop Surg Res.

[2] Zuo KK, Qin W, Miao Y, Zhu L. Analysis of risk factors of axial neck pain in posterior cervical single-door laminoplasty from the perspective of cervical sagittal plane. Frontiers in Surgery. 2022;9. 10.3389/fsurg.2022.973924 PMC9515390 36189387

[3] LI Z, Jiang J, Tian W, HE D, Mao J, Liu B, et al. Risk factors for axial symptoms after posterior cervical double door laminoplasty. Chinese Journal of Orthopaedics. 2018:1009–15.

